# A protocol for a systematic review of knowledge translation strategies in the allied health professions

**DOI:** 10.1186/1748-5908-6-58

**Published:** 2011-06-02

**Authors:** Shannon D Scott, Lauren Albrecht, Kathy O'Leary, Geoff DC Ball, Donna M Dryden, Lisa Hartling, Anne Hofmeyer, C Allyson Jones, Kathy Kovac Burns, Amanda S Newton, David Thompson, Terry P Klassen

**Affiliations:** 1 Faculty of Nursing, University of Alberta, Edmonton, Alberta, Canada; 2 Department of Pediatrics, Faculty of Medicine and Dentistry, University of Alberta, Edmonton, Alberta, Canada; 3 Department of Agricultural, Food and Nutritional Science, Faculty of Agricultural, Life & Environmental Science, University of Alberta, Edmonton, Alberta, Canada; 4 Pediatric Centre for Weight and Health, Stollery Children's Hospital, Edmonton, Alberta, Canada; 5 Evidence-based Practice Centre, University of Alberta, Edmonton, Alberta, Canada; 6 Alberta Research Centre for Health Evidence, University of Alberta, Edmonton, Alberta, Canada; 7 School of Nursing and Midwifery, University of South Australia, Adelaide, South Australia, Australia; 8 Department of Physical Therapy, Faculty of Rehabilitation Medicine, University of Alberta, Edmonton, Alberta, Canada; 9 Health Sciences Council, University of Alberta, Edmonton, Alberta, Canada; 10 Glenrose Rehabilitation Hospital, Edmonton, Alberta, Canada; 11 Women and Children's Health Research Institute, Edmonton, Alberta, Canada; 12 Stollery Children's Hospital, Edmonton, Alberta, Canada; 13 Northern Ontario School of Medicine, Thunder Bay, Ontario, Canada; 14 Manitoba Institute of Child Health, Department of Pediatrics and Child Health, University of Manitoba; 15 Winnipeg Regional Health Authority, Winnipeg, Manitoba, Canada

## Abstract

**Background:**

Knowledge translation (KT) aims to close the gap between knowledge and practice in order to realize the benefits of research through (a) improved health outcomes, (b) more effective health services and products, and (c) strengthened healthcare systems. While there is some understanding of strategies to put research findings into practice within nursing and medicine, we have limited knowledge of KT strategies in allied health professions. Given the interprofessional nature of healthcare, a lack of guidance for supporting KT strategies in the allied health professions is concerning. Our objective in this study is to systematically review published research on KT strategies in five allied health disciplines.

**Methods:**

A medical research librarian will develop and implement search strategies designed to identify evidence that is relevant to each question of the review. Two reviewers will perform study selection and quality assessment using standard forms. For study selection, data will be extracted by two reviewers. For quality assessment, data will be extracted by one reviewer and verified by a second. Disagreements will be resolved through discussion or third party adjudication. Within each profession, data will be grouped and analyzed by research design and KT strategies using the Effective Practice and Organisation of Care Review Group classification scheme. An overall synthesis across professions will be conducted.

**Significance:**

A uniprofessional approach to KT does not represent the interprofessional context it targets. Our findings will provide the first systematic overview of KT strategies used in allied health professionals' clinical practice, as well as a foundation to inform future KT interventions in allied healthcare settings.

## Background

On national and international stages, strategies to close the gap between what we "know" (research) and what we "do" (practice) have been consistently identified as a priority [1,2]. Knowledge translation (KT) aims to close the gap between knowledge and practice in order to realize the benefits of research in the areas of improved health outcomes, more effective health services and products, and strengthened healthcare systems [3]. It is well established that healthcare decisions based on sound evidence are crucial for ensuring high-quality patient care, optimal health outcomes, and quality and safety in healthcare systems. Thus, there is a need to identify and implement strategies that facilitate evidence-based decision making and ensure uptake of evidence into practice [4].

Over the past decade there has been rapid expansion of available scientific evidence to inform healthcare practices, with endorsement of evidence-informed healthcare by professional governing bodies and healthcare professional training programs. Concomitantly, improvements to the healthcare system have consistently been moving away from episodic unidisciplinary healthcare to interprofessional, collaborative models of practice with a patient-centered view of care.

Despite the interest in KT, there is a widening gap between research and practice, with the majority of healthcare professionals not drawing upon the best research evidence to guide clinical practice decisions [5,6]. In order to address this gap, various KT strategies have been developed and implemented, such as interactive educational sessions and audit and feedback of healthcare practices. Previous systematic reviews have evaluated the effectiveness of some of these KT strategies in the context of specific professional groups, such as physicians and nurses [7-10], yet existing reviews have two notable limitations. First, current reviews do not reflect the interprofessional and interdisciplinary flavor of Canada's healthcare landscape. There is a lack of systematic reviews specific for KT strategies for health professions allied to medicine and nursing. Health professional groups differ widely in training, education, organizational structure, and scope of practice and knowledge, thus, KT strategies that are successful in one profession may not seamlessly transfer to another profession. Effective healthcare delivery is dependent on a coordinated effort amongst health professionals from different disciplines [4,11,12], thus, a better understanding of effective KT strategies specific to health professions allied to medicine and nursing is urgently needed. Second, existing reviews privilege studies employing trial designs or studies with controls. This project will address these limitations through a series of interrelated systematic reviews (one per profession), synthesizing evidence from research using diverse study designs evaluating KT strategies in the allied health professions.

An allied health professional is an individual who provides specific types of acute care, management for chronic conditions, prevention, or health promotion and who is educated and licensed with credentials to provide that care but is not a physician, nurse, or dentist [13]. Each year, new disciplines are identified under the allied health umbrella; therefore, defining it is challenging [14]. For the purposes of this project, we have conceptualized allied health professionals to encompass and reflect the key health professions (allied to medicine and nursing) in the Canadian acute care health context, that is, rehabilitation medicine (physiotherapy, occupational therapy, speech-language pathology), dietetics, and pharmacy. These professions, as well as nursing and medicine, reflect the composition of many interprofessional teams providing healthcare in many Canadian acute care settings.

Understanding the most effective ways of translating evidence into clinical practice for different health professional groups and different healthcare settings was recognized as a national key synthesis priority in *Listening for Direction II *[1]. This priority (captured in *Managing and Adapting to Change) *highlights the need to break down organizational and professional silos that characterize healthcare and understand the most effective ways of translating evidence into practice from the perspective of health professional groups. The findings from this research project will provide critical information for three stakeholder groups: (1) allied health professionals, (2) decision makers who are charged with increasing the use of the latest research in healthcare settings, and (3) researchers conducting KT intervention studies.

One of the contributions of this project will be the development of products outlining the synthesis of findings in allied health profession-specific reviews. By synthesizing the evidence across the disciplines, in other words, reflecting an interprofessional flavor, we will begin to understand how the nature (*e.g*., extent of autonomy, scope of practice) and structure of the work within each healthcare discipline shapes the success of specific KT strategies. The involvement of allied healthcare decision makers from the clinical practice, research, and education sectors from inception of the project will ensure that the findings from this project result in the development of salient interprofessional evidence-based strategies to improve KT across multiple allied health professions, thereby reflecting the current healthcare landscape.

## Methods/design

### Aim and objectives

KT interventions or strategies are overt activities or devices that facilitate or encourage use of the research to make a clinical practice change. The literature has a great number of different strategies that can be used when introducing innovations and changes, such as reminders, financial incentives, organizational measures, audit, and feedback. This project will synthesize the evidence on KT interventions used in the allied healthcare professions. In order to do this, we will conduct a series of reviews for each profession using the same methods for each review. Following the completion of the five systematic reviews, we will synthesize the evidence across the reviews to look for similarities and patterns in terms of key factors, such as KT strategies employed, project design, and outcome measures.

The objectives for the large project are to

1. systematically locate, assess, and report on studies from each respective allied health profession [1] that have investigated the effects of KT interventions;

2. evaluate the interventions used to translate research into practice in terms of changes at the healthcare system, health provider, and/or patient level;

3. describe how the interventions worked and the modifying variables relevant to the respective context (that is, for whom does the intervention work, under what circumstances, and in what manner) [15];

4. provide possible strategies to facilitate KT for allied healthcare professionals and decision makers responsible for policy and institution/unit protocols in healthcare settings;

5. offer guidance for KT researchers in terms of the development of KT interventions for interprofessional healthcare teams.

### Key questions

In accordance with the larger project's aim and objectives, the following questions will guide this project:

1. What is the state of the science for KT strategies used in the allied healthcare professions?

2. What methodological approaches have been utilized in studies exploring KT strategies in the allied healthcare professions?

## Methods

The systematic reviews will follow a comprehensive process using rigorous methodological guidelines to synthesize diverse forms of research evidence [16]. While the methods outlined below are largely shaped by the conventional approach to systematic reviews, we will supplement these methods to accommodate the nature of the literature (*e.g*., complex interventions) and the different study designs (*e.g*., randomized controlled trials [RCTs], qualitative studies). We acknowledge that there is controversy about the legitimacy and feasibility of combining the findings of research studies employing different research methods [17] (*e.g*., qualitative and quantitative); however, the exclusive reliance on studies employing RCTs, controlled clinical trials, controlled before and after studies, or interrupted time series designs has created growing unease, particularly in the policy sector, about the utility of these types of systematic reviews [16]. In short, Cochrane-style reviews alone are not sufficient to reflect the complexities and intricacies of what decision makers consider relevant "evidence" to guide decision making. Thus, in order to be responsive to the needs of decision makers, the complexities of the clinical practice landscape, and the diversity in the KT literature, the methodological assumption guiding this project is one of inclusivity.

### Literature search

A research librarian, in collaboration with the research team, will develop and implement search strategies (Additional File 1) designed to identify evidence that is relevant to each question of the review. Our search for studies to be included in this review will be informed by previous systematic review work by members of this group (*e.g*., SDS and DT). Based on this work [10], we will work with a research librarian to refine and test our search strategy parameters for a project of this magnitude, involving a comprehensive set of subject headings and keywords that will be used in a variety of databases. Using language (English) and date (1985-2009) restrictions, we will systematically search the following electronic databases that store resources with this focus: PubMED, Scopus, Ovid MEDLINE, Cochrane Central Register of Controlled Trials, Cochrane Database of Systematic Reviews, Database of Abstracts of Reviews of Effects, Health Technology Assessment Database, HealthStar, EMBASE (Excerpta Medica), CINAHL (Cumulative Index to Nursing and Allied Health Literature), PsycINFO (Psychological Abstracts), ERIC, and Sociological Abstracts. We will also identify relevant dissertations and search the reference lists of included studies for relevant citations and will hand-search key journals and conference proceedings for the past five years from each of the allied health professions included in the project. Utilizing parameters from previous work [10] adapted for professional group nuances, we preliminarily searched three key databases (casting a wide net) to assess the scope of the literature to be screened for inclusion.

### Study inclusion criteria

Studies will not be excluded based upon research design. While controversial, the inclusion of study designs other than exclusively RCT and quasi-experimental is particularly important in an emerging field such as KT in the allied health professions. The merit in including these designs is that the results will reflect the rich and emerging literature base in KT strategies as well as generate hypotheses that could be tested in studies with more rigorous designs. Study selection will be guided by the inclusion criteria (Table 1).

**Table 1:** Inclusion criteria

Study design	Research studies including experimental, quasi-experimental, and nonexperimental designs (*e.g*., case study)
**Participants**	Occupational therapists, physical therapists, pharmacists, dietitians, speech-language pathologists

**Interventions**	Interventions/strategies with a primary purpose of translating research (or enhancing research uptake) into clinical practice; examples of potential interventions include reminders, use of multidisciplinary teams, educational programs, researcher-clinician interventions

**Outcomes**	Empirically assessed change (by way of quantitative or qualitative data) at the professional (*e.g*., change in clinical practice intervention), patient (*e.g*., improved response to the clinical practice intervention), or economic (*e.g*., change in staff-patient ratio) level

### Study selection

A two-step process will be used for study screening. First, two project reviewers will independently screen the titles and abstracts (when available) to determine whether a study meets the general inclusion criteria. Each article will be rated as Include, Exclude, or Unclear. The full text of all articles classified as Include or Unclear will be retrieved for formal review. Next, two reviewers will independently assess each study using a standard form that outlines the predetermined inclusion criteria. Disagreements will be resolved by discussion between the two reviewers or third party adjudication.

### Quality criteria

The process for assessing the methodological quality of included studies will be guided by study design. Two reviewers will independently assess the quality of included studies. Discrepancies in quality assessment will be resolved through discussion or third party adjudication. Interrater agreement will be calculated using the weighted kappa statistic [18]. The methodological quality of included quantitative studies will be assessed using the Quality Assessment Tool for Quantitative Studies (Additional File 2) [19]. The results from the tool will lead to an overall methodological rating of strong, moderate, or weak in eight sections: selection bias, study design, confounders, blinding, data collection methods, withdrawals/dropouts, intervention integrity, and analysis. This tool has been evaluated for content and construct validity and interrater reliability and meets accepted standards [19]. The methodological quality of qualitative studies will be assessed using the Quality in Qualitative Evaluation Framework (Additional File 3) [20]. This established framework assesses 18 aspects, including (1) credibility of the findings, (2) defensibility of the research design, (3) sample composition, (4) data sources, and (5) linkages between data, interpretation, and conclusions.

### Data extraction

Study data will be extracted using standard forms (Additional File 4) and entered into Microsoft Excel (Microsoft Corporation, Redmond, WA, USA) spreadsheets in tabular form. Data will be extracted by one reviewer and checked for accuracy and completeness by a second reviewer. Data to be extracted include study design and process, participant characteristics, KT intervention/strategy details, and study findings. The data extraction form will be tried on 10 studies to refine the form and ensure the form captures all of the intricacies of both qualitative and quantitative designs.

### Data analysis and synthesis

First, analysis will occur on a profession-by-profession basis, and a scholarly paper will document the findings for each profession. Study data for each profession will be grouped and analyzed by study design (*e.g*., qualitative, RCT, controlled before and after studies). Next, data will be aggregated and analyzed according to the type of KT intervention strategy or strategies within each allied health professional discipline. From this analysis we will present a descriptive analysis of the included studies and look at the patterns in terms of the success of the KT interventions.

A qualitative review of the studies across KT interventions will allow us to not only examine what strategies are successful but evaluate what it is about the different strategies that may work, for whom, and under what circumstances [15]. The value of our review is that data will not be pooled just to get an overall assessment of whether something could work but rather assess whether it does work given different scenarios. The results of our review will richly add to the evidence base as it goes beyond the results of a "typical" systematic review through the inclusion of all study designs.

Following the analysis of the evidence within each profession, the evidence across the professions will be synthesized to reflect the interprofessional nature of Canada's healthcare landscape. Descriptive analyses will be conducted to look for patterns in terms of successful KT interventions. Evidence tables will be created that describe all of the studies included in this project. Variables to be evaluated in the descriptive analysis include country of primary author, study design, quality assessment of studies, and KT intervention outcomes.

if there is sufficient clinical and statistical homogeneity across groups of studies employing RCT designs, we will perform meta-analyses using Review Manager (The Cochrane Collaboration, Copenhagen, Denmark) [21]. We will first calculate the overall intervention effects using DerSimonian and Laird's random effects model [22,23]. This model assumes there is a different underlying effect for each study and takes this into consideration as an additional source of variation, leading to wider (more conservative) confidence intervals. We will use the inverse of the variances for each study to weight its intervention effect in the pooled analysis (thereby assigning greater weight to larger studies, with a more precise effect size estimate). For continuous data, we will present analyses as weighted mean differences with 95% confidence intervals; for dichotomous data, we will present data as pooled odds ratios using an inverse variance method with 95% confidence intervals. A test for heterogeneity will be conducted to determine the degree of similarity in the studies' outcomes. For each pooled analysis, we will assess statistical heterogeneity using the Cochrane chi-square test [24] with conventional statistical significance (*p *< .05) and the I^2 ^statistic, which describes the percentage of total variation across studies that is due to heterogeneity rather than chance [25]. We will explore sources of heterogeneity through subgroup analyses (*e.g*., for interventions and each allied healthcare profession).

### Integrated knowledge translation plan

#### Decision maker and stakeholder partnerships

Our multidisciplinary team of KT researchers, systematic review experts, clinicians, and decision makers is based on the linkage and exchange model [26]. All team members will participate in regularly scheduled teleconferences focusing on project progress and discussion of project findings. Also, to ensure that our synthesis outputs respond to the information needs of stakeholders, we have developed an Advisory Panel that the research team will engage as needed to provide strategic advice in terms of interfacing with national and international allied health professionals and organizations. The Advisory Panel is comprised of international expert allied health professionals who will advise the research team on the development of the across-profession synthesis and on the strategic development of suitable "end products" of the systematic reviews for planned dissemination to the appropriate local, national, and international groups and associations.

In addition to having an engaged Advisory Panel to facilitate strategic networking and dissemination of findings from the systematic reviews, our research team will have ongoing consultations with national allied health associations. Through our initial consultations with national professional organizations, we were able to identify stakeholder preference for one page e-formats for our research outputs. Furthermore, at the local level, we have the support of two key interprofessional organizations. Our collaborative interactions with national allied health associations, the development and engagement of an interprofessional Advisory Panel, the hands-on involvement of decision makers on our research team, and support from key interprofessional venues ensure that the findings are policy relevant and that recommendations are appropriate and achievable in the clinical, educational, and policy sectors.

Meaningful engagement with decision makers by means of our Advisory Panel and our ongoing consultations with stakeholders from national allied health organizations will ensure that (a) our research questions and project aims are relevant from the outset and applicable to issues of concerns to them locally, (b) project funds are used judiciously, and (c) the findings inform innovative strategies to make a quality difference in clinical practice, education, and research endeavors within the allied health professions.

#### Dissemination of our findings

Knowledge sharing between decision makers, clinicians, and researchers is increasingly important to ensure that practice and policy are based on sound evidence. Our knowledge-dissemination plan is based on the Canadian Health Services Research Foundation Model [27] and the research of Lavis and colleagues [28]. We will customize the research results to targeted user groups: allied health practitioners, decision makers, and KT researchers.

We will disseminate the findings from our systematic review to allied healthcare professionals in ways that are congruent with our systematic review findings (*e.g*., using interactive educational meetings). We will work closely with the national organizations of each allied healthcare professional group to transfer our findings in venues and formats that reflect disciplinary preferences. We will also work with the media to prepare and distribute media releases of our findings.

In order to disseminate our findings to decision makers, we will present at healthcare research seminars and conferences, provide specific fact sheets, and meet face to face or by phone to discuss the findings from this systematic review with those decision makers interested in adopting strategies to enhance the utilization of research in practice. We will highlight practical strategies that could maximize transfer of useful findings into their specific setting. We will also circulate a one-page executive summary and project technical report that addresses the project's objectives. As indicated in our lett**e**rs of support, our Advisory Panel members are committed to assisting with the KT process to ensure that the information we present face to face and in the fact sheets is tailored to the specific audience and incorporates practical implementation strategies. We will summarize our research findings in lay language (*e.g*., executive summary, brief report) thus demonstrating our clear commitment to ensuring that our project findings are transferable and used by various individuals from a wide range of disciplines and sectors (*e.g*., clinical, research, education, policy). These project outputs will use illustrations, color, and appealing packaging to ensure that the products are engaging. Furthermore, key messages and recommendations will be emphasized to ensure that the users know how to take concrete action. We will include real-world examples and use arts-based techniques such as storytelling to bring the research findings to life.

We will use both traditional and innovative mechanisms for disseminating results to other KT researchers. We will present at conferences, publish in relevant peer-reviewed journals, and post fact sheets on websites specifically aimed at knowledge utilization and transfer.

## Summary

To date, systematic reviews on KT strategies have largely had a unidisciplinary focus [7-10] primarily due to rigid systematic review protocols that privilege RCT designs. The drawback of these reviews is that they do not reflect the vital interdisciplinary nature of the healthcare landscape. This interprofessional review will serve as a state of the science on KT strategies used in allied health professionals' clinical practice. This project will allow us to (a) make interprofessional comparisons across the reviews in this proposal, (b) recommend transfer of effective KT strategies from one profession to another, (c) develop concrete recommendations with and for application by allied health professionals and decision makers at various levels of the healthcare system interested in enhancing outcomes through the application of research, and (d) identify areas of investigation for researchers designing interprofessional KT intervention studies.

## Competing interests

The authors declare that they have no competing interests.

## Authors' contributions

SDS conceptualized this study and secured study funding from the Canadian Institutes for Health Research (CIHR). She lead and designed this study. LA and KO coordinated the study team and the study itself. Both assisted with the study design. The remaining authors all assisted with the study design and are listed alphabetically. In addition, DMD and LH provided methodological consultation. As well, KKB was the principal knowledge user for this study. All authors read and approved the final manuscript.

## Supplementary Material

Additional File 1 **Preliminary search strategy Initial search strategy developed by medical research librarian**. Click here for file

Additional File 2 **Quality assessment tool for quantitative studies Tool to be used to assess methodological quality of included quantitative research studies**. Click here for file

Additional File 3 **Quality assessment tool for qualitative studies Tool used to be used to assess methodological quality of included qualitative research studies**. Click here for file

Additional File 4 **Data extraction form Standard form to be used to extract data from included studies**. Click here for file
