# Supplementary material for: A protocol for a systematic review of knowledge translation strategies in the allied health professions
Source: Implement Sci. 2011 Jun 2;6:58. doi: 10.1186/1748-5908-6-58 (PMC3130686; doi:10.1186/1748-5908-6-58)
Supplement: Additional File 1 — Preliminary search strategy Initial search strategy developed by medical research librarian. [file 1748-5908-6-58-S1.PDF]

Additional File 1 - Preliminary search strategy

CINAHL (1982-April 2008)

1. exp (name of discipline, e.g., physiotherapy)/
2. exp (name of clinicians, e.g., physiotherapists)/
3. exp Practice Guidelines/
4. exp AUDIOVISUALS/
5. exp PAMPHLETS/
6. exp "POLICY AND PROCEDURE MANUALS"/
7. exp Protocols/
8. exp Staff Development/
9. inservice$.mp.
10. exp "Seminars and Workshops"/
11. exp Education, Clinical/
12. exp Staff Development Instructors/
13. exp (name of discipline, e.g., physiotherapy) Consultants/
14. (chang$ adj2 agent$).mp.
15. (facilitat$ adj2 change$).mp.
16. (coordinat$ adj2 change$).mp.
17. exp Quality Assurance/
18. (critical adj1 appraisal).mp.
19. exp Quality Improvement/
20. exp Reminder Systems/
21. (champion$ adj1 change$).mp.
22. exp "Diffusion of Innovation"/
23. exp (name of discipline, e.g., physiotherapy) Practice, Research-Based/
24. evidence based (name of discipline, e.g., physiotherapy).mp.
25. (utilizat$ or utilisa$ or uptake or transfer$ or implement$ or disseminat$ or diffusion$ or translat$).mp.
26. journal club.mp.
27. exp (name of discipline, e.g., physiotherapy) Practice, Evidence-Based/
28. 1 or 2
29. or/3-21
30. 29 or 26
31. or/22-25
32. 31 or 27
33. 28 and 30 and 32
34. limit 31 to research

Medline (1966-April 2008)

1. exp (name of discipline, e.g., physiotherapy)/
2. exp (name of clinicians, e.g., physiotherapists)/
3. exp Practice Guidelines/
4. exp AUDIOVISUAL AIDS/

5. exp PAMPHLETS/
6. exp MANUALS/
7. exp CLINICAL PROTOCOLS/
8. exp Inservice Training/
9. seminar.mp.
10. workshop.mp.
11. clinical education.mp.
12. exp (name of discipline, e.g., physiotherapy)/Clinicians/
13. staff instructor$.mp.
14. exp Consultants/
15. (chang$ adj2 agent$).mp.
16. (facilitator$ adj2 chang$).mp.
17. (coordinator$ adj2 chang$).mp.
18. (champion$ adj2 chang$).mp.
19. journal club.mp.
20. exp Quality Assurance, Health Care/
21. exp REMINDER SYSTEMS/
22. exp "Diffusion of Innovation"/
23. exp Evidence-Based Medicine/
24. exp (name of discipline, e.g., physiotherapy)/Research/
25. (utilizat$ or utlisat$ or uptake or transfer$ or implement$ or disseminat$ or diffusion$ or translat$).mp.
26. 1 or 2
27. or/3-21
28. or/22-25
29. 26 and 27 and 28

PsychINFO (1887-April 2008)

1. exp (name of discipline, e.g., physiotherapy)/
2. exp (name of clinician, e.g., physiotherapists)/
3. exp Treatment Guidelines/
4. exp EDUCATIONAL AUDIOVISUAL AIDS/
5. pamphlets.mp.
6. (policy and procedure).mp. [mp=title, abstract, subject headings, table of contents, key concepts]
7. protocol.mp.
8. exp Professional Development/
9. inservice.mp.
10. workshop.mp.
11. seminar.mp.
14. instructor.mp.
15. (name of discipline, e.g., physiotherapy)/consultant.mp.
16. (chang$ adj2 agent$).mp.
17. (facilitat$ adj2 chang$).mp.
18. (coordinat$ adj2 change).mp.

19. exp "Quality of Services"/
20. (critical adj1 appraisal).mp.
21. reminder$.mp.
22. (champion$ adj1 change$).mp.
23. diffusion of innovation.mp.
24. exp Decision Making/
25. (research and (utiliz$ or utilis$ or uptake or transfer or implement$ or disseminat$ or translat$)).mp. [mp=title, abstract, subject headings, table of contents, key concepts]
26. (knowledge and (utiliz$ or utilis$ or uptake or transfer or implement$ or disseminat$ or translat$)).mp. [mp=title, abstract, subject headings, table of contents, key concepts]
27. (evidence adj1 practice).mp.
28. journal club.mp.
29. 1 or 2
30. or/2-22
31. 30 or 28
32. or/23-27
33. 29 and 31 and 32

HealthSTAR/Non-medline (1975-April 2008)

1.exp (name of discipline, e.g., physiotherapy)
2. exp (name of clinician, e.g., physiotherapist)
3. exp Practice Guidelines/
4. exp AUDIOVISUAL AIDS/
5. exp PAMPHLETS/
6. exp MANUALS/
7. exp CLINICAL PROTOCOLS/
8. exp Inservice Training/
9. seminar.mp.
10. workshop.mp.
11. clinical education.mp.
12. staff instructor$.mp.
13. exp Consultants/
14. exp (name of discipline, e.g., physiotherapy)/Clinicians/
15. (chang$ adj2 agent$).mp.
16. (facilitator$ adj2 chang$).mp.
17. (coordinator$ adj2 chang$).mp.
18. (champion$ adj2 chang$).mp.
19. journal club.mp.
20. exp Quality Assurance, Health Care/
21. exp REMINDER SYSTEMS/
22. exp "Diffusion of Innovation"/
23. exp Evidence-Based Medicine/
24. exp (name of discipline, e.g., physiotherapy)/Research/

25. (utilizat$ or utlisat$ or uptake or transfer$ or implement$ or disseminat$ or diffusion$ or translat$).mp.
26. 1 or 2
27. or/3-21
28. or/22-25
29. 26 and 27 and 28
30. limit 29 to non-medline

ERIC (1966-April 2008)

1. (name of discipline, e.g., physiotherapy)/*.tx
2. (name of clinicians, e.g., physiotherapist).tx
3. (practice guidelines).tx
4. audiovisual.tx
5. (policy and procedure).tx
6. protocol*.tx
7. (staff development).tx
8. (in service).tx
9. seminar.tx
10. workshop.tx
11. (journal club).tx
12. (clinical education).tx
13. instructor.tx
14. consultant.tx
15. (change agent).tx
16. champion.tx
17. coordinator.tx
18. facilitator.tx
19. (clinical educator).tx
20. (quality assurance).tx
21. (critical appraisal).tx
22. (quality improvement).tx
23. (reminder).tx
24. or/3-23
25. 1 or 2 and 24

Scopus (1960-March 2009)

(((TITLE-ABS-KEY(**"diffusion of innovation"**))) OR ((((TITLE-ABS-KEY(**knowledge** W/3 **utiliz***))) OR ((TITLE-ABS-KEY(**knowledge** W/3 **utilis***))) OR ((TITLE-ABS-KEY(**knowledge** W/3 **uptake**))) OR ((TITLE-ABS-KEY(**knowledge** W/3 **transfer**))) OR ((TITLE-ABS-KEY(**knowledge** W/3 **implement***))) OR ((TITLE-ABS-KEY(**knowledge** W/3 **adoption**)))) OR (((TITLE-ABS-KEY(**knowledge** W/3 **translat***))) OR ((TITLE-ABS-KEY(**knowledge** W/3 **usage**))) OR ((TITLE-ABS-KEY(**knowledge** W/3 **shar***))) OR ((TITLE-ABS-KEY(**knowledge** W/3 **diffusion**))))) OR ((((TITLE-ABS-KEY(**research** W/3 **utiliz***))) OR ((TITLE-ABS-KEY(**research** W/3 **utilis***))) OR ((TITLE-ABS-KEY(**research** W/3 **uptake**))) OR ((TITLE-ABS-KEY(**research** W/3 **transfer**))) OR ((TITLE-ABS-KEY(**research** W/3 **implement***))) OR ((TITLE-ABS-KEY(**"research use"**))) OR ((TITLE-ABS-KEY(**research** W/3 **adoption**)))) OR (((TITLE-ABS-KEY(**research** W/3 **translat***))) OR ((TITLE-ABS-KEY(**research** W/3 **usage**))) OR ((TITLE-ABS-KEY(**research** W/3 **shar***))) OR ((TITLE-ABS-KEY(**research** W/3 **diffusion**))))) OR ((((TITLE-ABS-KEY(**evidence** W/3 **utiliz***))) OR ((TITLE-ABS-KEY(**evidence** W/3 **utilis***))) OR ((TITLE-ABS-KEY(**evidence** W/3 **uptake**))) OR ((TITLE-ABS-KEY(**evidence** W/3 **transfer**))) OR ((TITLE-ABS-KEY(**evidence** W/3 **implement***))) OR ((TITLE-ABS-KEY(**evidence** W/3 **adoption**)))) OR (((TITLE-ABS-KEY(**evidence** W/3 **translat***))) OR ((TITLE-ABS-KEY(**evidence** W/3 **usage**))) OR ((TITLE-ABS-KEY(**evidence** W/3 **shar***))) OR ((TITLE-ABS-KEY(**evidence** W/3 **diffusion**)))))) AND (TITLE-ABS-KEY(**physiotherap***)) AND (((TITLE-ABS-KEY(**"journal club"**)) OR ((TITLE-ABS-KEY(**"practice guideline"** OR **"practice guidelines"**)) OR ((TITLE-ABS-KEY((**audio** W/3 **instruction***) OR (**auditory** W/3 **instruction***)) OR TITLE-ABS-KEY((**audio** W/3 **teach***) OR (**auditory** W/3 **teach***)))) OR ((TITLE-ABS-KEY((**audio** W/3 **aide***) OR (**auditory** W/3 **aide***)) AND TITLE-ABS-KEY(**teach*** OR **instruction***))) OR ((TITLE-ABS-KEY((**visual** W/3 **aide***)) AND TITLE-ABS-KEY(**teach*** OR **instruction***))) OR (TITLE-ABS-KEY((**visual** W/3 **instruction**) OR (**visual** W/3 **teach***))) OR (TITLE-ABS-KEY(**pamphlet***)) OR (TITLE-ABS-KEY(**manual***)) OR ((TITLE-ABS-KEY(**treatment** W/0 **protocol***) OR TITLE-ABS-KEY(**clinical** W/0 **protocol***)))) OR ((TITLE-ABS-KEY(**"staff development"** OR **"staff training"** OR **"employee development"** OR **"employment training"**)) OR (TITLE-ABS-KEY(**inservice**)) OR (TITLE-ABS-KEY(**seminar*** OR **workshop** OR **"clinical education"** OR **"continuing education"**)) OR ((TITLE-ABS-KEY(**change*** W/2 **agent***) OR TITLE-ABS-KEY(**facilitat*** W/2 **change***))) OR ((TITLE-ABS-KEY(**coordinat*** W/2 **change***) OR TITLE-ABS-KEY(**co-ordinat*** W/2 **change***))) OR (TITLE-ABS-KEY(**"quality assurance"**)) OR (TITLE-ABS-KEY(**critical** W/1 **appraisal**))) OR ((TITLE-ABS-KEY(**"quality improvement"**)) OR (TITLE-ABS-KEY(**"reminder systems"**)) OR (TITLE-ABS-KEY(**champion*** W/1 **change***)))))
