# Supplementary material for: A protocol for a systematic review of knowledge translation strategies in the allied health professions
Source: Implement Sci. 2011 Jun 2;6:58. doi: 10.1186/1748-5908-6-58 (PMC3130686; doi:10.1186/1748-5908-6-58)
Supplement: Additional File 2 — Quality assessment tool for quantitative studies Tool to be used to assess methodological quality of included quantitative research studies. [file 1748-5908-6-58-S2.PDF]

Additional File 2 – *Quality assessment tool for quantitative studies*

QUALITY ASSESSMENT TOOL FOR QUANTITATIVE STUDIES

COMPONENT RATINGS

A) SELECTION BIAS

(Q1) Are the individuals selected to participate in the study likely to be representative of the target population?

Very likely
Somewhat likely
Not likely
Can't tell

(Q2) What percentage of selected individuals agreed to participate?

80 - 100% agreement
60 – 79% agreement
less than 60% agreement
Not applicable
Can't tell

**RATE THIS SECTION**	**STRONG**	**MODERATE**	**WEAK**
See dictionary	1	2	3

B) STUDY DESIGN

Indicate the study design

Randomized controlled trial
Controlled clinical trial
Cohort analytic (two group pre + post)
Case-control
Cohort (one group pre + post (before and after))
Interrupted time series
Other specify ____________________________
Can't tell

Was the study described as randomized? If NO, go to Component C.

No Yes

If Yes, was the method of randomization described? (See dictionary)

No Yes

If Yes, was the method appropriate? (See dictionary)

No Yes

No Yes **RATE THIS SECTION**	**STRONG**	**MODERATE**	**WEAK**
**See dictionary**	1	2	3

C) CONFOUNDERS

(Q1) Were there important differences between groups prior to the intervention?

Yes
No
Can't tell

The following are examples of confounders:

Race
Sex
Marital status/family
Age
SES (income or class)
Education
Health status
Pre-intervention score on outcome measure

(Q2) If yes, indicate the percentage of relevant confounders that were controlled (either in the design (e.g. stratification, matching) or analysis)?

80 – 100% (most)
60 – 79% (some)
Less than 60% (few or none)
Can't Tell

**RATE THIS SECTION**	**STRONG**	**MODERATE**	**WEAK**
**See dictionary**	1	2	3

D) BLINDING

(Q1) Was (were) the outcome assessor(s) aware of the intervention or exposure status of participants?

Yes
No
Can't tell

(Q2) Were the study participants aware of the research question?

Yes
No
Can't tell

**RATE THIS SECTION**	**STRONG**	**MODERATE**	**WEAK**
**See dictionary**	1	2	3

E) DATA COLLECTION METHODS

(Q1) Were data collection tools shown to be valid?

Yes
No
Can't tell

(Q2) Were data collection tools shown to be reliable?

Yes
No
Can't tell

**RATE THIS SECTION**	**STRONG**	**MODERATE**	**WEAK**
**See dictionary**	1	2	3

F) WITHDRAWALS AND DROP-OUTS

(Q1) Were withdrawals and drop-outs reported in terms of numbers and/or reasons per group?

Yes
No
Can't tell
Not Applicable (i.e. one time surveys or interviews)

(Q2) Indicate the percentage of participants completing the study. (If the percentage differs by groups, record the lowest).

80 -100%
60 - 79%
less than 60%
Can't tell
Not Applicable (i.e. Retrospective case-control)

RATE THIS SECTION	STRONG	MODERATE	WEAK	
**See dictionary**	1	2	3	Not Applicable

G) INTERVENTION INTEGRITY

(Q1) What percentage of participants received the allocated intervention or exposure of interest?

80 -100%
60 - 79%
less than 60%
Can't tell

(Q2) Was the consistency of the intervention measured?

Yes
No
Can't tell

(Q3) Is it likely that subjects received an unintended intervention (contamination or co-intervention) that may influence the results?

Yes
No
Can't tell

H) ANALYSES

(Q1) Indicate the unit of allocation (circle one)
community organization/institution practice/office individual

(Q2) Indicate the unit of analysis (circle one)
community organization/institution practice/office individual

(Q3) Are the statistical methods appropriate for the study design?

Yes
No
Can't tell

(Q4) Is the analysis performed by intervention allocation status (i.e. intention to treat) rather than the actual intervention received?

Yes
No
Can't tell

GLOBAL RATINGS

COMPONENT RATINGS
Please transcribe the information from the gray boxes on pages 1-4 onto this page. See dictionary on how to rate this section.

**A**	**Selection Bias**	**Strong**	**Moderate**	**Weak**	
		1	2	3	
**B**	**Study Design**	**Strong**	**Moderate**	**Weak**	
		1	2	3	
**C**	**Confounders**	**Strong**	**Moderate**	**Weak**	
		1	2	3	
**D**	**Blinding**	**Strong**	**Moderate**	**Weak**	
		1	2	3	
**E**	**Data Collection Method**	**Strong**	**Moderate**	**Weak**	
		1	2	3	
**F**	**Withdrawals and Dropouts**	**Strong**	**Moderate**	**Weak**	
		1	2	3	Not Applicable

GLOBAL RATING FOR THIS PAPER (circle one):

1	STRONG	(no WEAK ratings)
2	MODERATE	(one WEAK rating)
3	WEAK	(two or more WEAK ratings)

With both reviewers discussing the ratings:

Is there a discrepancy between the two reviewers with respect to the component (A-F) ratings?

No Yes

If yes, indicate the reason for the discrepancy

1 Oversight
2 Differences in interpretation of criteria
3 Differences in interpretation of study

Final decision of both reviewers (circle one):

1 STRONG
2 MODERATE
3 WEAK
