# Supplementary material for: A protocol for a systematic review of knowledge translation strategies in the allied health professions
Source: Implement Sci. 2011 Jun 2;6:58. doi: 10.1186/1748-5908-6-58 (PMC3130686; doi:10.1186/1748-5908-6-58)
Supplement: Additional File 3 — Quality assessment tool for qualitative studies Tool used to be used to assess methodological quality of included qualitative research studies. [file 1748-5908-6-58-S3.PDF]

Additional File 3 – *Quality assessment tool for qualitative studies*

FRAMEWORK FOR ASSESSING QUALITATIVE EVALUATIONS

Study being appraised

____

Findings	A) Appraisal questions	B) Quality indicators (possible features for consideration)	C) Notes on study being appraised
**1**	How credible are the findings?	Findings/conclusions are supported by data/study evidence (*i.e.. the reader can see how the researcher arrived at his/her conclusion; the 'building blocks' of analysis and interpretation are evident)*<br><br>Findings/conclusions 'make sense'/have a coherent logic<br><br>Findings/conclusions are resonant with other knowledge and experience (*this might include peer or member review)*<br><br>Use of corroborating evidence to support or refine findings (*i.e. other data sources have been used to examine phenomena; other research evidence has been evaluated: see also Q14*)	
**2**	How has knowledge/understanding been extended by the research?	Literature review (where appropriate) summarizing knowledge to date/key issues raised by previous research<br><br>Aims and design of study set in the context of existing knowledge/understanding; identifies new areas for investigation (*i.e. in relation to policy/practice/substantive theory)*<br><br>Credible/clear discussion of how findings have contributed to knowledge and understanding (*i.e. of the policy, programme or theory being reviewed;:*might be applied to new policy developments, practice or theory<br><br>Findings presented or conceptualized in a way that offers new insights/alternative ways of thinking<br><br>Discussion of limitations of evidence and what remains unknown/unclear or what further	

Findings	A) Appraisal questions	B) Quality indicators (possible features for consideration)	C) Notes on study being appraised
		information/research is needed	
**3**	How well does the evaluation address its original aims and purpose?	Clear statement of study aims and objectives; reasons for any changes in objectives<br><br>Findings clearly linked to the purposes of the study – and to the initiative or policy being studied<br><br>Summary or conclusion directed towards aims of study<br><br>Discussion of limitations of study in meeting aims (*i.e. are there limitations because of restricted access to study settings or participants, gaps in the sample coverage, missed or unresolved areas of questioning; incomplete analysis; time constraints?*)	
**4**	Scope for drawing wider inference – how well is this explained?	Discussion of what can be generalized to wider population from which sample is drawn/case selection has been made<br><br>Detailed description of the contexts in which the study was conducted to allow applicability to other settings/contextual generalities to be assessed<br><br>Discussion of how hypotheses/propositions/findings may relate to wider theory; consideration of rival explanations<br><br>Evidence supplied to support claims for wider inference (*either from study or from corroborating sources*)<br><br>Discussion of limitations on drawing wider inference (*i.e. re-examination of sample and any missing constituencies: analysis of restrictions of study settings for drawing wider inference*)	
**5**	How clear is the basis of evaluative appraisal?	Discussion of how assessments of effectiveness/evaluative judgments have been reached (*i.e. whose judgments are they and on what basis have they been reached?*)	

Findings	A) Appraisal questions	B) Quality indicators (possible features for consideration)	C) Notes on study being appraised
		Description of any formalized appraisal criteria used, when generated and how and by whom they have been applied<br><br>Discussion of the nature and source of any divergence in evaluative appraisals<br><br>Discussion of any unintended consequences of intervention, their impact and why they arose	
**6**	How defensible is the research design?	Discussion of how overall research strategy was designed to meet aims of study<br><br>Discussion of rationale for study design<br><br>Convincing argument for different features of research design (*i.e. reasons given for different components or stages of research; purpose of particular methods or data sources, multiple methods, time frames etc.*)<br><br>Use of different features of design/data sources evident in findings presented<br><br>Discussion of limitations of research design and their implications for the study evidence	
**7**	How well defended is the sample design/target selection of cases/documents?	Description of study locations/areas and how and why chosen<br><br>Description of population of interest and how sample selection relates to it (*i.e. typical, extreme case, diverse constituencies etc.*)<br><br>Rationale for basis of selection of target sample/settings/documents (*i.e. characteristics/features of target sample/settings/documents, basis for inclusions and exclusions, discussion of sample size/number of cases/setting selected etc.*)<br><br>Discussion of how sample/selections allowed required comparisons to be made	
**8**	Sample composition/case inclusion – how well is the eventual coverage described?	Detailed profile of achieved sample/case coverage<br><br>Maximizing inclusion (*i.e. language matching or*	

Findings	A) Appraisal questions	B) Quality indicators (possible features for consideration)	C) Notes on study being appraised
		translation; specialized recruitment; organized transport for group attendance)<br><br>Discussion of any missing coverage in achieved samples/cases and implications for study evidence *(i.e. through comparison of target and achieved samples, comparison with population etc.*)<br><br>Documentation of reasons for non-participation among sample approached/non-inclusion of selected cases/documents<br><br>Discussion of access and methods of approach and how these might have affected participation coverage	
**9**	How well was the data collection carried out?	Discussion of:<br>• Who conducted data collection<br>• Procedures/documents used for collection/recording<br>• Checks on origin/status/authorship of documents<br><br>Audio or video recording of interviews/discussions/conversations (*If not recorded, were justifiable reasons given?*)<br><br>Description of conventions for taking field notes (*i.e. to identify what form of observations were required/ to distinguish description from researcher commentary/analysis*)<br><br>Discussion of how fieldwork methods or settings may have influenced data collected<br><br>Demonstration through portrayal and use of data, that depth, detail and richness were achieved in collection	
**10**	How well has the approach to, and formulation of, the analysis been conveyed?	Description of form of original data (*i.e. use of verbatim transcripts, observation or interview notes, documents, etc.*)<br><br>Clear rationale for choice of data management method/tool/package<br><br>Evidence of how descriptive analytic categories, classes, labels etc. have been generated and used	

Findings	A) Appraisal questions	B) Quality indicators (possible features for consideration)	C) Notes on study being appraised
		(*i.e. either through explicit discussion or portrayal in the commentary*)<br><br>Discussion, with examples, of how any <u>constructed</u> analytic concepts/typologies etc. have been devised and applied	
**11**	Contexts of data source – how well are they retained and portrayed?	Description of background or historical developments and social/organizational characteristics of study sites or settings<br><br>Participants' perspectives/observations placed in personal context (*i.e. use of case studies/vignettes/individual profiles, textual extracts annotated with details of contributors*)<br><br>Explanation or origins/history of written documents<br><br>Use of data management methods that preserve context (*facilitate within case description and analysis*	
**12**	How well has diversity of perspective and content been explored?	Discussion of contribution of sample design/case selection in generation diversity<br><br>Description and illumination of diversity/multiple perspectives/alternative positions in the evidence displayed<br><br>Evidence of attention to negative cases, outliers or exceptions<br><br>Typologies/models of variation derived and discussed<br><br>Examination of origins/influences on opposing or differing positions<br><br>Identification of patterns of association/linkages with divergent positions/groups	
**13**	How well has detail, depth and complexity (i.e. richness) of the data been conveyed?	Use and exploration of contributors' terms, concepts and meanings<br><br>Unpacking and portrayal of nuance/subtlety/intricacy within data	

Findings	A) Appraisal questions	B) Quality indicators (possible features for consideration)	C) Notes on study being appraised
		Discussion of explicit and implicit explanations<br><br>Detection of underlying factors/influences<br><br>Identification and discussion of patterns of association/conceptual linkages within data<br><br>Presentation of illuminating textual extracts/observations	
**14**	How clear are the links between data, interpretation and conclusions - i.e. how well can the route to any conclusions be seen?	Clear conceptual links between analytic commentary and presentations of original data (*i.e. commentary and cited data relate; there is an analytic context to cited data, not simply repeated description*)<br><br>Discussion of how/why particular interpretation/significance is assigned to specific aspects of data – with illustrative extracts of original data<br><br>Discussion of how explanations/ theories/conclusions were derived – and how they relate to interpretations and content of original data (*i.e. how warranted*); whether alternative explanations explored<br><br>Display of negative cases and how they lie outside main proposition/theory/hypothesis etc; or how proposition etc. revised to include them	
**15**	How clear and coherent is the reporting?	Demonstrates link to aims of study/research questions<br><br>Provides a narrative/story or clearly constructed thematic account<br><br>Has structure and signposting that usefully guide reader through the commentary<br><br>Provides accessible information for intended target audiences(s)<br><br>Key messages highlighted or summarized	
**16**	How clear are the assumptions/theoretical perspectives/values that have shaped the form and output of	Discussion/evidence of the main assumptions/hypotheses/theoretical ideas on which the evaluation was based and how these affected the form coverage or output of the evaluation (*the*	

Findings	A) Appraisal questions	B) Quality indicators (possible features for consideration)	C) Notes on study being appraised
	the evaluation?	*assumption here is that no research is undertaken without some underlying assumptions or theoretical ideas*)<br><br>Discussion/evidence of the ideological perspectives/values/philosophies of research team and their impact on the methodological or substantive content of the evaluation (*again, may not be explicitly stated*)<br><br>Evidence of openness to new/alternative ways of viewing subject/theories/assumptions (*i.e. discussion of learning/concepts/constructions that have emerged from the data; refinement restatement of hypotheses/theories in light of emergent findings; evidence that alternative claims have been examined*)<br><br>Discussion of how error or bias may have arisen in design/data collection/analysis and how addressed, if at all<br><br>Reflections on the impact of the researcher on the research process	
**17**	What evidence is there of attention to ethical issues?	Evidence of thoughtfulness/sensitivity about research contexts and participants<br><br>Documentation of how research was presented in study settings to participants (*including, where relevant, any possible consequences of taking part*)<br><br>Documentation of consent procedures and information provided to participants<br><br>Discussion of confidentiality of data and procedures for protecting<br><br>Discussion of how anonymity of participants/sources was protected<br><br>Discussion of any measures to other information/advice/services etc. at end of study (*i.e. where participation exposed the need for these*)<br><br>Discussion of potential harm or difficulty through participation, and how avoided	
**18**	How adequately has the	Discussion of strengths and weaknesses of data	

Findings	**A) Appraisal questions**	**B) Quality indicators (possible features for consideration)**	**C) Notes on study being appraised**
	research process been documented?	sources and methods<br><br>Documentation of changes made to design and reasons; implications for study coverage<br><br>Documentation and reasons for changes in sample coverage/data collection/analytic approach; implications<br><br>Reproduction of main study documents (*i.e. letters of approach, topic guides, observation templates, data management, frameworks, etc.*)	
