# Supplementary material for: A protocol for a systematic review of knowledge translation strategies in the allied health professions
Source: Implement Sci. 2011 Jun 2;6:58. doi: 10.1186/1748-5908-6-58 (PMC3130686; doi:10.1186/1748-5908-6-58)
Supplement: Additional File 4 — Data extraction form Standard form to be used to extract data from included studies. [file 1748-5908-6-58-S4.PDF]

Additional File 4 – Data extraction form

Reviewer		Date
**Systematic Review Of KT Strategies Used In The Allied Health Professions Extraction Tool For Included Studies – Draft**		
**Publication Information**		
Study	First Author	
Date	Journal	
Country		
**Demographics**		
Sample Size	Type Of Sample	
Design Of Study		
**Data Analysis**		
Statistical Tests Used		
Effect Of Intervention		
Qualitative Data Analysis		
Results		
**Theoretical Framework For Study**	Done (Specify Which Theory)	Not Done

**Intervention/Implementation**:		
**Type Of Intervention** (circle only one, append additional pages if more than one intervention)		
**Professional**	**Financial**	**Organizational**
• educational materials	• provider interventions	• revision of professional roles
• local consensus processes	• patient interventions	• multidisciplinary teams
• educational outreach visits	•	• formal integration of services
• local opinion leaders	•	• introduction of new role
• patient-mediated interventions	•	• skill mix changes
• audit and feedback	•	• continuity of care changes
• reminders	•	• interventions to boost morale
• marketing	•	• communication/case discussion
• mass media	•	•
• clinical practice guideline	•	•
•	•	•
**Patient-oriented**	**Structural**	**Regulatory**
• participation in governance	• setting of service delivery changes	• changes in practitioner liability

• mail order pharmacies	• physical structure changes	• patient complaint management
• suggestions/complaints strategy	• medical records systems changes	• peer review
•	• service benefits changes	• licensure
•	• quality monitoring organization	•
•	• facility ownership changes	•
•	• staff organization	•
•	•	•

What Was The Focus Of The Intervention?

Did It Involve A Physical Component?

Format Of Intervention

Interpersonal	Paper	Audio/Visual	Computer/Interactive	Multiple Media Used	Not Clear	Other

Recipient Of Intervention

Individual(Type)	Group (Type)	Not Clear	Other

Deliverer Of Intervention (Circle All Appropriate)

Local Expert	Researcher	Management	Educator	Not Clear	

Control Groups

No Intervention	Standard Practice	Control Group Received Intervention After Follow-Up	Other Intervention (Type)

Outcomes

Type Of Targeted Behaviour (Circle All That Apply)

Health Promotion	Illness Prevention	Illness Management	Ordering	Procedures	Patient Education
Professional/Patient Communication	Documentation	Resource Use	Patient Outcomes (Specify)		
System Outcomes (Specify)		Not Clear	Other (Specify)		

How Outcomes Were Measured:

Frequency Of Outcome Measurement:

Length Of Follow-Up Period:

Length Of Time Between End Of Intervention And Beginning Of Follow-Up/Outcome Measurement:

Additional Notes:
